# B Cells Isolated from Individuals Who Do Not Respond to the HBV Vaccine Are Characterized by Higher DNA Methylation-Estimated Aging Compared to Responders

**DOI:** 10.3390/vaccines12080880

**Published:** 2024-08-02

**Authors:** Katarzyna Malgorzata Kwiatkowska, Simona Anticoli, Stefano Salvioli, Luciano Calzari, Davide Gentilini, Christian Albano, Reparata Rosa Di Prinzio, Salvatore Zaffina, Rita Carsetti, Anna Ruggieri, Paolo Garagnani

**Affiliations:** 1 Department of Medical and Surgical Sciences (DIMEC), University of Bologna, 40126 Bologna, Italy; stefano.salvioli@unibo.it; 2 Istituto Superiore di Sanità, Center for Gender Specific Medicine, 00161 Rome, Italy; simona.anticoli@iss.it (S.A.); anna.ruggieri@iss.it (A.R.); 3 IRCCS Azienda Ospedaliero, Universitaria di Bologna, 40138 Bologna, Italy; 4 Bioinformatics and Statistical Genomics Unit, Istituto Auxologico Italiano IRCCS, 20095 Cusano Milanino, Italy; l.calzari@auxologico.it (L.C.);; 5 Department of Brain and Behavioral Sciences, Università di Pavia, 27100 Pavia, Italy; 6 Immunology Research Area, B Cell Unit, Ospedale Pediatrico Bambino Gesù IRCCS, 00146 Rome, Italyrita.carsetti@opbg.net (R.C.); 7 Occupational Medicine/Health Technology Assessment and Safety Research Unit, Clinical-Technological Innovations Research Area, Ospedale Pediatrico Bambino Gesù IRCCS, 00146 Rome, Italysalvatore.zaffina@opbg.net (S.Z.)

**Keywords:** hepatitis B, vaccine, health care workers, sex, immune response, B lymphocytes, DNA methylation clock, epigenetic clock, epigenetic age, aging

## Abstract

Healthcare workers (HCWs) are a high-risk group for hepatitis B virus (HBV) infection. Notably, about 5–10% of the general population does not respond to the HBV vaccination. In this study, we aimed to investigate DNA methylation (DNAm) in order to estimate the biological age of B cells from HCW of both sexes, either responder (R) or non-responder (NR), to HBV vaccination. We used genome-wide DNA methylation data to calculate a set of biomarkers in B cells collected from 41 Rs and 30 NRs between 22 and 62 years old. Unresponsiveness to HBV vaccination was associated with accelerated epigenetic aging (DNAmAge, AltumAge, DunedinPoAm) and was accompanied by epigenetic drift. Female non-responders had higher estimates of telomere length and lower CRP inflammation risk score when compared to responders. Overall, epigenetic differences between responders and non-responders were more evident in females than males. In this study we demonstrated that several methylation DNAm-based clocks and biomarkers are associated with an increased risk of non-response to HBV vaccination, particularly in females. Based on these results, we propose that accelerated epigenetic age could contribute to vaccine unresponsiveness. These insights may help improve the evaluation of the effectiveness of vaccination strategies, especially among HCWs and vulnerable patients.

## 1. Introduction

Hepatitis B virus (HBV) infection is still regarded as a serious global health concern, affecting millions of individuals worldwide, despite vaccination campaigns carried out in the majority of nations. An estimated 296 million people worldwide had chronic HBV infection in 2019, which was responsible for 820,000 viral hepatitis-related deaths, mostly because of the development of cirrhosis and hepatocellular carcinoma [1]. Due to their frequent contact with blood products and other body fluids, primarily through needle-stick injuries, healthcare workers (HCWs) have a four-fold higher risk of contracting HBV infection than the general population. Up to 3 million HCWs are exposed to hepatitis B virus every year and about 66,000 develop infections [2]. Thus, HBV vaccination is highly recommended for HCWs in most countries, including Italy, to protect against the virus [3,4].

Despite the immunogenicity of the HBV vaccine, in 5–10% of subjects, the anti-HBs antibody titer remains below the protective level (≥10 mIU/mL). The vaccinees unable to reach the protective antibody threshold after two vaccination cycles are considered “non-responders” [5,6,7,8,9]. A greater understanding of the factors affecting vaccine responsiveness offers opportunities to improve vaccine immunogenicity and efficacy and to optimize immunization strategies. This is particularly important for at-risk subjects and vulnerable individuals, such as those diagnosed with diabetes, obesity, HIV, HCV, celiac disease, inflammatory bowel disease, or other conditions. There is solid evidence that fundamental intrinsic factors, such as age and sex [9,10,11], have a strong influence on the HBV vaccine response. Male sex was associated with an increased incidence of unresponsiveness to HBV vaccination in a large retrospective study on healthy individuals and HCWs (16–70 years old) [12]. However, it is not yet clear how sex and age interact in modulating HBV vaccine response.

Epigenetic clock are models built on DNA methylation (DNAm) data designed to capture various aspects of aging and changes in biological processes that occur throughout life. They are associated with different biological hallmarks of aging [13]. The methylation drift concept embraces naturally occurring age-related alterations within the methylome that are environmental and random effects [14]. This phenomenon manifests across the population as continuously increasing dysregulation of methylation patterns with age [15,16]. Therefore, these DNAm-based estimates are interesting and promising candidates to be investigated in the context of responsiveness to HBV vaccination. As far as we know, there are still no studies related to epigenetic aging and the effectiveness of immune protection after the HBV vaccine.

Given (i) the importance of the effective prevention of HBV infection (especially in at-risk HCWs and vulnerable groups), (ii) the non-negligible rate of vaccine unresponsiveness among individuals who have received HBV vaccine, and (iii) the potential role of age- and sex-related epigenetic biomarkers linked to this phenomenon, we have investigated five estimates of biological aging, predicted using DNA methylation data of isolated B cells from a cohort of 71 healthcare workers, aged between 22 and 62 years. These individuals received HBV vaccination and either developed insufficient or protective immune responses according to anti-HB titer.

### Aim

In this study, we intend to identify potential general and sex-specific changes in DNAm-based estimates of biological aging in B cells that may be associated with the responsiveness to HBV vaccination.

## 2. Materials and Methods

### 2.1. Cohort 

Study participants were recruited among the healthcare workers employed at the Bambino Gesù Children’s Hospital (Rome, Italy). All enrolled individuals (*n* = 74) were subjected to the mandatory HBV vaccination, according to the national prevention strategy regulated by the Italian law n. 165/1991 [4] and underwent periodic health surveillance for assessment of anti-HBV protection through anti-HBs titers. The exclusion criteria were: (1) being born to a HbsAg+ mother; (2) being infected with the hepatitis virus and (3) not being vaccinated. Appropriate signed informed consent was obtained from each subject prior to enrollment. This study was approved by the Ethical Committee of Istituto Superiore di Sanità (AOO-ISS 09/05/2021–0017778). B cell donors were assigned to the responder group (R; *n* = 44) based on anti-HB titers ≥10 mIU/mL. HCWs were selected to the non-responder group (NR; *n* = 30) if they had antibody levels of <10 mIU/mL after the primary vaccination, and their titer did not increase after a second vaccination cycle remaining <10 mIU/mL. Antibody levels were measured 1 month after the last dose of the second cycle.

### 2.2. Biological Material, DNA Extraction, Bisulfite Conversion

PBMC samples were collected from the recruited vaccinated subjects and B cells were isolated by negative selection using the RosetteSep™ Human B Cell Enrichment Cocktail (Stemcell Technologies, Vancouver, BC, Canada). Genomic DNA was extracted with QIAamp DNA Blood Mini Kit (Qiagen, Venlo, The Netherlands) following the manufacturer’s “Blood or Body Fluid Spin Protocol”, using for final elution 130 μL of Buffer AE. Samples were quantified on a Qubit fluorometer using a dsDNA Broad Range Assay kit (Thermo Fisher Scientific, Waltham, MA, USA), normalized with H_2_O to contain 1200 ng of DNA in a final volume of 50 μL and bisulfite-converted with EZ-96 DNA Methylation Kit Deep-Well (Zymo Research, Orange, CA, USA), as indicated in the producer’s instructions. DNA methylation was assessed using Infinium Human MethylationEPIC BeadChip (Illumina, San Diego, CA, USA) according to the standard protocol. Samples were accurately randomized in all the processing steps.

### 2.3. DNA Methylation Data Preprocessing

All the file handling and preprocessing steps were run in R (v3.6.3) on Linux environment. We used the minfi R Bioconductor package to parse raw idat files produced on Illumina platform and to extract signal intensities in the green and red channels. Per each array probe, we calculated detection *p*-value and we performed quality control, eventually excluding low-quality samples with mean detection *p*-value above 0.05 and removing the probes that failed in at least one of the samples. The raw values of intensities in green and red channels were normalized using *Noob* (normal-exponential out-of-band) background correction method with dye-bias normalization dedicated to Illumina Infinium methylation arrays [17]. Eventually we transformed methylation data to Beta-values that were used in calculation of DNAm-based markers.

### 2.4. Epigenetic Age Estimation of DNAm-Based Biomarkers

In the studied cohort, we assessed a selection of DNA methylation-based (DNAm) biomarkers of biological aging published in the up-to-date literature: (i) pan-tissue Horvath’s DNAmAge clock (DNAmAge) [13], (ii) estimator of telomere length (DNAmTL) [18], (iii) predictor of pace of aging (DunedinPoAm) [19], (iv) pan-tissue DNA-methylation epigenetic clock based on deep learning (AltumAge) [20] and (v) CRP-associated methylation risk score based on DNA methylation signature of chronic low-grade inflammation as measured by serum levels of C-Reactive protein CRP (CRP_CpG_risk_score) [21]. Additionally, in order to validate the original outcomes, we used bolstered models proposed by Levine and colleagues (PC-clocks) to calculate estimates of original DNAmAge and DNAmTL markers improved with principal component analysis (PCDNAmTL and PCHorvath2) [22]. We estimated DNAmAge and DNAmTL with New DNA Methylation Age Calculator (available at https://dnamage.genetics.ucla.edu/; accessed on 20 October 2023). We used the DunedinPoAm38 R package to compute pace of aging in our array data. AltumAge was calculated using Python script available online: https://github.com/rsinghlab/AltumAge (accessed on 27 November 2023). In order to calculate CRP_CpG_risk_score and PC-clocks, we used R scripts provided by their authors in GitHub repositories: https://github.com/Mwielscher/EWAS_CRP and https://github.com/MorganLevineLab/PC-Clocks respectively (accessed on 27 March 2023).

### 2.5. Statistical Analysis

Prior to evaluating the differences between two phenotypic groups, the eventual outliers were removed, i.e., samples for which DNAm-based estimates were below Q1—1.5IQR or above Q3 + 1.5IQR, where Q1—first quartile, Q3—third quartile, and IQR—interquartile range. In order to identify the links between patients’ phenotype (responder or non-responder to HBV vaccine) and biological aging, we performed two-stage residual-outcome regression analysis. Specifically, for each of epigenetic biomarkers, a reference linear regression model describing its relationship with chronological age was built, using data from the responder group as control. The created model was then applied to R and NR groups to predict the surrogate under investigation and calculate the chronological age-corrected residuals. Finally, the differences between residuals of two phenotypic groups were assessed with parametric Student’s *t*-test, and the Benjamini–Hochberg (BH) procedure was used to perform multiple hypothesis testing correction. The approach described above was applied (i) to the entire cohort adjusting linear regression model with sex (assigned biological attribute) covariate (allowing identification of tendencies in biological aging independent of person’s sex) and (ii) separately in female and male group (enabling determination of sex-specific patterns).

### 2.6. Distribution of Standard Deviation

Changes in epigenetic variance were assessed through computation of standard deviation for a total of 21,948 unique probes of the EPIC platform that embraced all CpG sites used in the estimation of five DNAm-based surrogates: DNAmAge, DNAmTL, DunedinPoAm, AltumAge and CRP_CpG_risk_score. The differences between R and NR samples were evaluated with a two-sample Kolmogorov–Smirnov (KS) test. The results were presented as density plots visualizing the distribution of standard deviation values after logarithmic transformation.

## 3. Results

### 3.1. Sample Processing

Experiments were performed on 74 samples (R = 44 and NR = 30) of B cells separated from PBMCs, with an average of 2.8 ± 1.8 × 10^6^ isolated cells per vial. Three samples did not pass the quality control of extracted genomic DNA due to a low quantity of DNA. Eventually, 71 samples (41 R and 30 NR) were eligible for bisulfite conversion and successfully underwent methylome profiling.

### 3.2. Cohort Characteristics

Overall, responders and non-responders were similar in the studied cohort for chronological age and number of cells in the samples used for genomic DNA extraction (Table 1). The female/male ratio was higher in R than NR group; however, this difference was not statistically significant (*p* = 0.179).

Similarly, considering female and male groups separately, no significant differences in the age of individuals and number of B cells were found between R and NR, as summarized in Table 2.

### 3.3. Biological Age Estimates in the Overall Responder/Non-Responder Population

**Figure 1:**
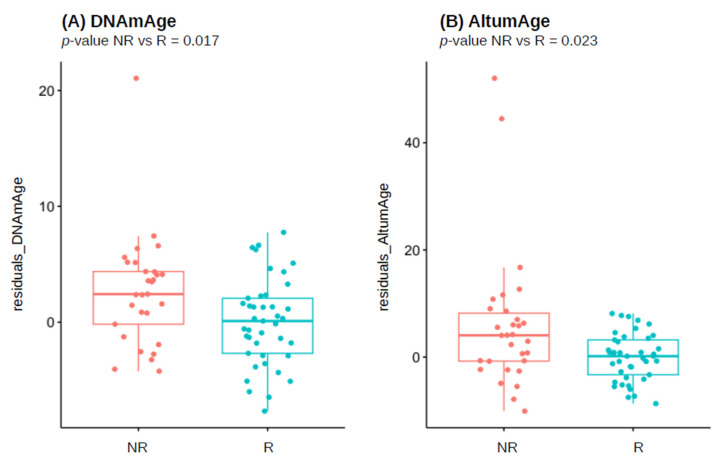
Boxplots presenting difference in residuals of: (**A**) Horvath’s DNAmAge, (**B**) AltumAge between responders (R) and non-responders (NR) of entire studied cohort, after adjustment for chronological age. Nominal *p*-values from 2SR analysis are reported.

The results of two-stage residual-outcome regression analysis of selected DNAm-based biomarkers in the overall population showed that, in general, NRs are biologically older than Rs, as summarized in Table 3. We observed a statistically significant, sex-independent accelerated biological age measured with Horvath’s DNAmAge clock (nominal *p*-value = 0.017; Figure 1A) and pan-tissue AltumAge clock (nominal *p*-value = 0.023; Figure 1B) in NR when compared to R group. However, for both predictors, BH-adjusted *p*-values were not significant. PC-improved estimate of DNAmAge, PCHorvath1 clock, did not replicate the significant difference between NR and R groups. For the remaining DNAm-based surrogates (DNAmTL, DunedinPoAm and CRP_CpG_risk_score), we did not find any difference between the two groups.

### 3.4. Biological Age Estimates in the Female Responder/Non-Responder Population

**Figure 2:**
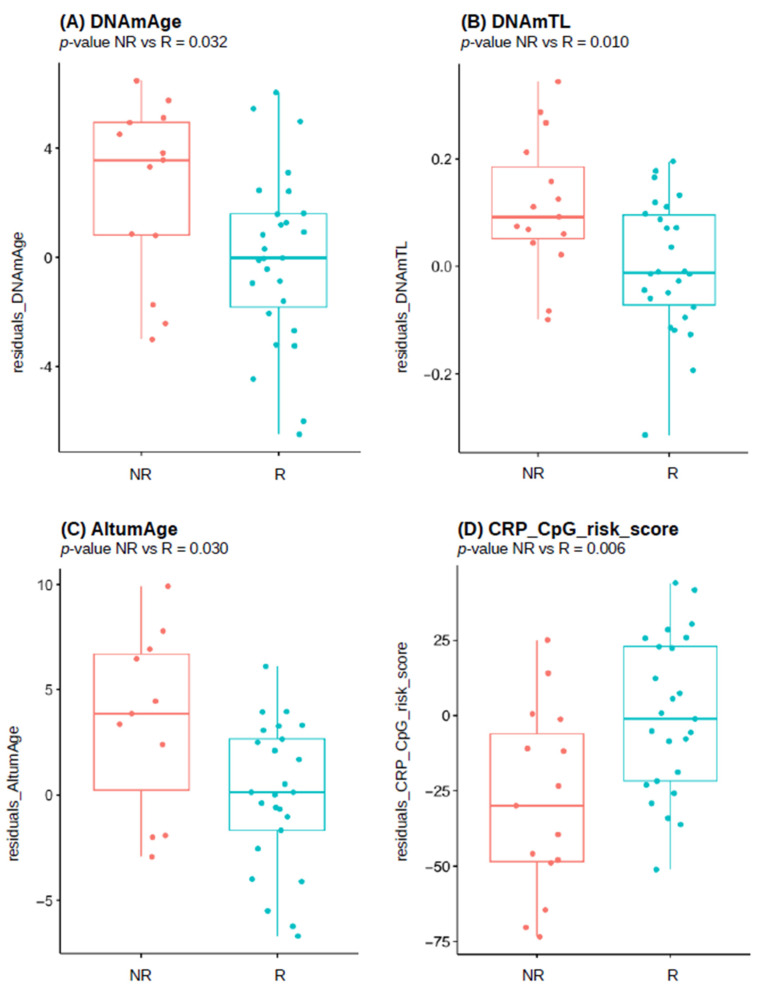
Boxplots presenting difference in residuals of: (**A**) Horvath’s DNAmAge, (**B**) DNAmTL, (**C**) AltumAge, (**D**) CRP_CpG_risk_score, between female responders (R) and non-responders (NR) of studied cohort, after adjustment for chronological age. Nominal *p*-values from 2SR analysis are reported.

The outcome of sex-specific analysis of DNAm-based estimates in females is presented in Table 4. Horvath’s DNAmAge clock was significantly higher in NR than R, reaching nominal *p*-value of 0.032 (Figure 2A). There was a significant difference in DNAm-based estimate of DNAmTL telomere length (nominal *p*-value = 0.010), suggesting that NR females are expected to have longer telomeres compared to the R group (Figure 2B). Both two biomarkers remained significant after BH correction for multiple tests with adjusted *p*-values of 0.045 and 0.028, respectively. Using the bolstered algorithms to estimate DNAmAge and DNAmTL PC-based versions, only the estimated length of telomeres resulted as significantly higher in the female NR group than in R. In contrast, the improved PCHorvath1 clock did not confirm previously found differences between responding and non-responding women.

Another measure of biological aging–DunedinPoAm predicting pace of aging did not reveal any significant difference between R and NR females. Scores obtained using AltumAge algorithm were significantly higher among the non-responder than in the responder group (nominal *p*-value 0.003; Figure 2C) and remainedsignificant after correction for multiple testing (adjusted *p*-value = 0.045).

Finally, CRP DNA methylation risk score, the marker for chronic low-grade inflammation, was significantly lower in female NR when compared to R (nominal *p*-value 0.006; Figure 2D) and the observed difference remained significant after correction for multiple tests (adjusted *p*-value = 0.028).

### 3.5. Biological Age Estimates in the Male Responder/Non-Responder Population

**Figure 3:**
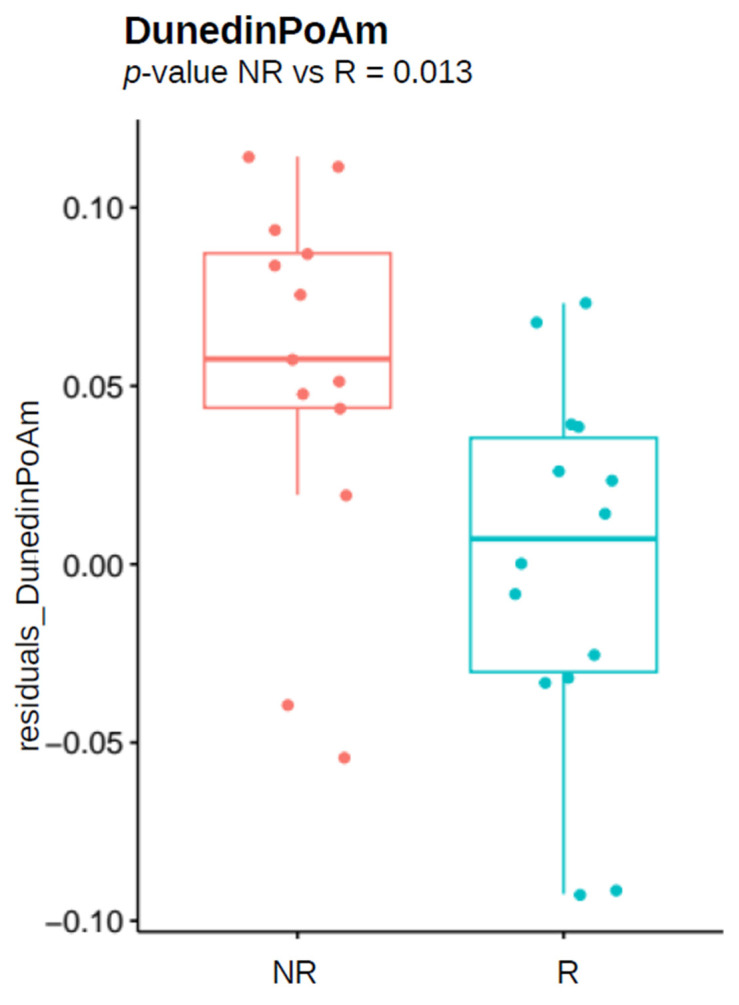
Boxplot presenting difference in residuals of DunedinPoAm estimate between male responders (R) and non-responders (NR) of studied cohort, after adjustment for chronological age. Nominal *p*-values from 2SR analysis are reported.

The differences between R and NR male HCWs in DNAm-based estimates were less evident than in females, as shown by the results summarized in Table 5. The only DNAm-based estimate that reached statistical significance was the DunedinPoAm marker (nominal *p*-value of 0.013), indicating higher pace of aging among male NR individuals compared to R (Figure 3). However, the variable did not remain significant after BH correction for multiple tests.

### 3.6. Epigenetic Entropy (Variance Estimation in DNA Methylation) in Responder/Non-Responder Population

**Figure 4:**
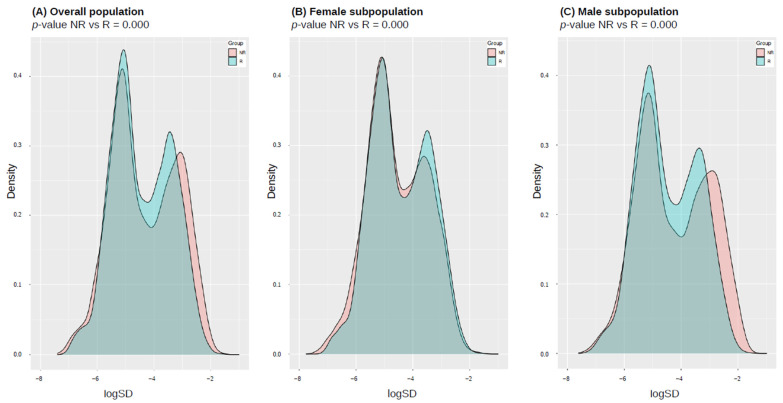
Density plots visualizing the distribution of standard deviation values in clock CpGs after logarithmic transformation in responders (R) and non-responders (NR) in (**A**) overall population, (**B**) female and (**B**,**C**) male subpopulations. *p*-values from two-sample Kolmogorov–Smirnov test are reported.

**Figure 5:**
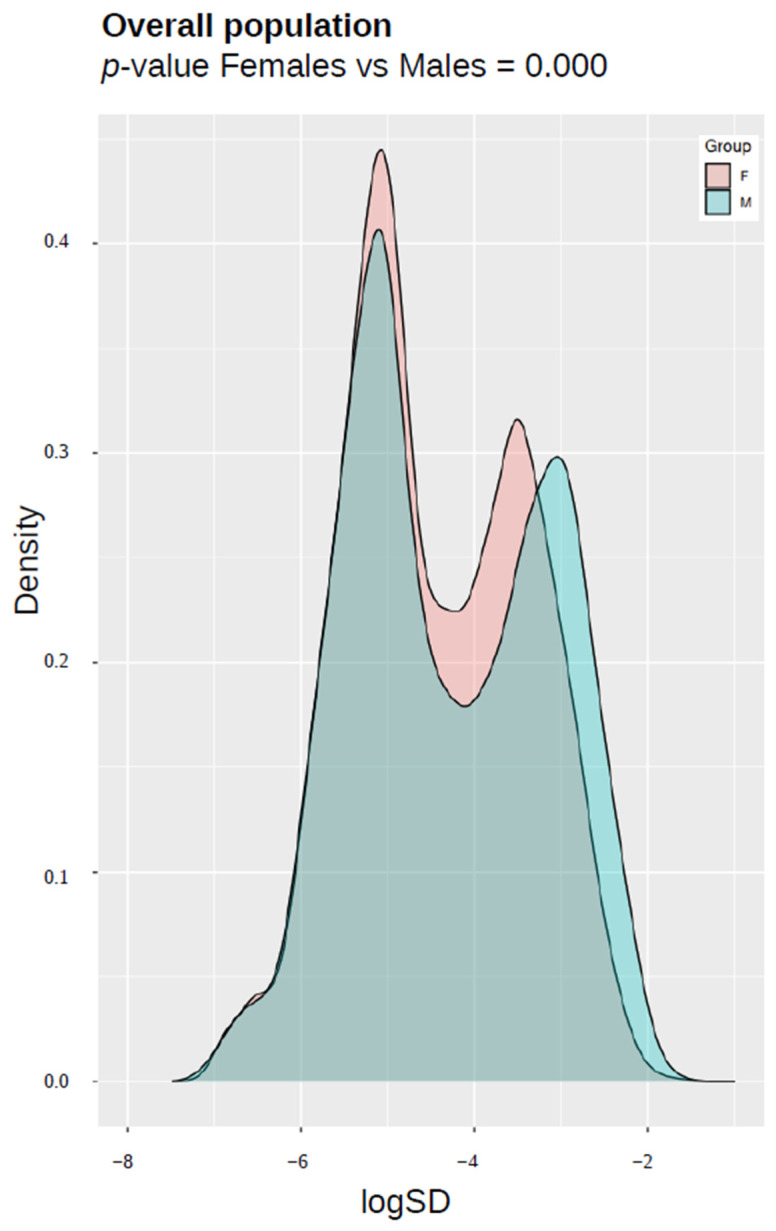
Density plots visualizing the distribution of standard deviation values after logarithmic transformation in female and male subpopulations. *p*-value from two-sample Kolmogorov–Smirnov test is reported.

Methylation standard deviation distribution analysis was performed in order to evaluate the phenotype- and sex-related epigenetic drift in CpGs associated with biological aging. We analyzed the variance across 21,948 unique probes used to calculate five epigenetic estimates in responding and non-responding HCWs, considering the entire cohort and each sex separately. Statistically significant differences in epigenetic drift were confirmed between the R and NR groups in both the combined sexes (*p*-value = 0.000; Figure 4A) and the sex-separated analysis (*p*-value = 0.000; Figure 4B,C). These alterations related to vaccine responsiveness were more pronounced in males than in females. Furthermore, when comparing the aggregated responders and non-responders in the male versus female groups, men showed significantly higher standard deviation of methylation data across clock-CpGs compared to women (*p*-value = 0.000; Figure 5).

## 4. Discussion

To the best of our knowledge, this is the first study on immune response to HBV vaccination that has been performed in B cells isolated from peripheral blood with a coverage of DNA methylation at whole genome level. The study population comprised a group of individuals at high risk for infection, such as HCWs. We have assessed a set of five DNAm-based biomarkers that have not been previously described in a similar setting: DNAmAge, DNAmTL, DunedinPoAm, AltumAge and CRP_CpG_risk_score. We extended the evaluation of biological aging, including examination of epigenetic drift—gradual deregulation of DNAm patterns with age, leading to deviations from a normal epigenetic state. Our experimental design included both sexes, thus making it possible to identify and characterize, for the first time, sex-specific patterns in DNAm aging that are associated with the response to HBV vaccine.

According to the results obtained in this study, unresponsiveness to HBV vaccination was correlated to the accelerated epigenetic clocks and faster rate of biological aging. We found that, in the overall population of HCWs, non-responder individuals had a significantly higher biological age estimated by pan-tissue clocks: DNAmAge and AltumAge, compared to successful responders. When considering sex groups separately, these changes resulted as even more pronounced in females, while in males a similar trend was present, although not statistically significant, with the exception of pace of aging.

The results showed a connection between a weakened response to HBV vaccination and epigenetic drift, which is an age-related global loss of homogeneity in DNA methylation patterns associated with an increased risk of disease. The NR group had significantly increased methylation variability in clock-CpG probes compared to responders, even when considering males and females separately.

Overall, the findings presented above suggest that a poor immune response to HBV vaccine is associated with increased biological age of B cells. This conclusion is not unexpected and is consistent with the generally accepted model of age-related decline of the immune function and the concomitant reduction of vaccination response with aging [23]. Several studies on the HBV vaccine have reported a reduced rate of immune response with increasing age of the vaccinees [24,25]. However, the most recent literature does not provide any additional data supporting a link between the effectiveness of vaccination response and the progression of biological age measured by DNAm-based estimates. In a previous study, we investigated the acceleration or deceleration of Horvath’s epigenetic clock in relation to the response to influenza vaccination. In the study, performed on total PBMCs, we did not find any difference between responders and non-responders [26]. Here, we analyzed isolated B cells, which are the cell type responsible for antibody production and represent only about 5% of the total PBMC population. Thus, the inconsistency between the two studies may be explained by the fact that B-cell and not PBMC biological age modulates the antibody response. Alternatively, different mechanisms may determine the ability to respond to HBV or influenza vaccines.

Telomere shortening occurs with aging and is associated with several pathological health conditions and diseases [27,28,29,30,31], therefore a longer telomere length (TL) should be associated with a younger age. However, nowadays the capability of TL to catch biological age is debated, since a growing body of results indicate that it has limited resolving power for aging quality [32]. Moreover, it has also been demonstrated that the different clocks are not always concordant between different cell types [33] and it is worthwhile remembering that DNAmTL is not a direct measurement of TL, but is an estimate based on DNA methylation. Having said that, this result is intriguing as it is possibly related to B responsiveness rather than biological age, due to the peculiar telomere processing in B cells during transition from naïve to memory phenotype. However, it is not clear which type of telomere modification may be associated with decreased responsiveness to vaccination in the B-cell compartment. At present, it has been reported that germinal center and memory B cells have longer telomeres with respect to naïve ones [34], while other authors reported that memory and naïve B cells do not display significant differences in TL [35] and, most of all, to the best of our knowledge, no data are available on DNAmTL in relation to B cells responsiveness. Our data suggest that elevated DNAmTL could be associated with vaccine unresponsiveness (even though only in females), but further studies are needed to clarify this neglected aspect, which should be relevant for the biology of acquired immunity and vaccination, especially in elderly persons.

Furthermore, our results demonstrated that female NRs had a reduced CRP DNA methylation risk score compared to same sex R individuals. This score is a DNAm-based signature of a low-grade inflammation as measured by C-Reactive protein. A lower, though not statistically significant, CRP DNA methylation score was also observed in male NR and the overall population. Low-grade inflammation may potentiate the immune response, thus explaining the lower score of the NR group. 

Lastly, it seems that vaccine response-related age acceleration measured by different methylation clocks is more pronounced in women rather than men. This result is, however, to be considered with caution, as it could be accounted for by different reasons: first of all, according to the results of standard deviation distribution analysis, the epigenetic drift, although observed in both sexes, was much more pronounced in males than in females, leading to a lower number of significant results in the former group. The trend towards higher age-related DNA methylation variability in males was previously observed in the population between 60 and 94 years [36]. Second, it must be noted that the lower numerosity of the male group might have led to less significant differences between R and NR subjects.

The strength of this study is the fact that it provided for the first time an in-depth, state-of-the-art analysis of DNA methylation patterns of purified B cells (not whole blood nor PBMCs) from both male and female responders and non-responders to the HBV vaccine. As a consequence, we reached an unprecedented level of complexity in the analysis of the results (clocks, predictors). This study can thus pave the way for more research, not only in terms of sex-based responses to vaccination but also in the field of epigenetics in sex medicine.

### Perspectives and Significance

In this study we have shown that DNAm-based clocks may contribute to the knowledge of the molecular mechanisms involved in and featuring HBV vaccine responsiveness. Despite the efficacy of the HBV vaccine, around 5–10% of the population does not develop protective responses after vaccination [37]. The elevated incidence of non- or poor response was found in groups diagnosed with diabetes, obesity, end-stage renal disease, HIV, HCV, celiac disease, and inflammatory bowel disease, among others [37,38]. The presence of any of these comorbidities in HCW increases the risk of insufficient immune response to HBV vaccination. Different strategies and improved vaccination protocols (for example, vaccine adjuvants, recombinant vaccines, and immune enhancement via up-regulation of dendritic cells) have been developed to meet the challenges of non-protective responses, and they can be now offered to the NR population to increase the immunogenicity of the HBV vaccine [39]. Our study contributes to this topic by offering: (i) a biomarker correlated with absent or poor immune response to HBV, and (ii) a novel biological mechanism (accelerated DNAm aging) to explain the reason for the reduced antibody response. This could be of great importance for HCWs, who have a high risk of exposure to HBV. Further studies are necessary to investigate the features of B-cell biological clocks in the elderly and vulnerable patients (such as immunocompromised or frail/multimorbid patients). Whereas biological clocks have been investigated in total blood cells, we concentrated our study on purified B cells. B cells play a fundamental role in the response to vaccination because they produce antibodies which protect the organism by neutralizing viruses and represent the most widely used biological read-out of vaccine immunogenicity. Our results suggest that a poor immune response to the HBV vaccine is associated with increased biological age of B cells. As the level of specific antibodies induced after vaccination is not routinely measured, we do not know whether the immune response to other vaccinations may be also impaired when B cells show signs of aging.

Unresponsiveness to HBV vaccination in cases without classical risk factors has often been assigned to genetic background. Genome-wide association studies (GWAS) in responders and non-responders to the HBV vaccine provided a number of single nucleotide polymorphisms (SNPs) linked to the immune response. Most of these publications identified genetic variants spread over the human leukocyte antigen–DP (HLA–DP) region [40,41,42,43,44,45], which is also correlated with susceptibility to chronic HBV infection. Moreover, although less prominent, single SNPs in FOXP1 [46] and ITGAL (CD11a) [47] have also been identified in the NR population. It will be important to complete our study with the genetic profiling of the NR participants in order to obtain insight into the interplay between genetic background and epigenetic modifications in determining the unresponsiveness to the HBV vaccine.

However, the present manuscript reports differences in DNA methylation-based biomarkers, which are models (particularly epigenetic clocks) created on correlation with chronological age and/or age-related health characteristics. For this reason, in general, they mirror overall condition of the body influenced by several risk factors, for example, obesity or smoking, hence they reflect the immunological function/dysfunction of the organism. As such, it seems reasonable to expect that the results presented on epigenetic aging in unresponsiveness to HBV vaccination could be valid also for other vaccines.

## 5. Conclusions

In this work, we have identified HBV vaccine responsiveness-related patterns of epigenetic aging and sex-specific DNA methylation biomarkers in isolated B cells. Our results suggest that B-cell aging may be associated with impairment of the antibody response. We present evidence, for the first time, of important epigenetic alterations underlying the ability of B cells to respond to the antigenic challenge of vaccination, indicating that the pace of aging in this cell type is correlated with vaccine response. Additionally, we have found evidence that biological age affects vaccine responsiveness in a different way between men and women. 

From our results, we can say that B cell epigenetic age could be considered a biomarker of unresponsiveness to HBV vaccine. Here we report that, among individuals comparable for chronological age, HBV non-responders show epigenetic signs of B cell aging. Our results suggest that biological clocks may be used to measure the B cell fitness and predict their ability to perform optimal immune responses. 

It is to remark that biological clocks have not been yet directly linked to specific functional alterations of proteomic pathways, and this limits at the moment the possibility to drive gene-specific strategy to counteract the age-dependent epigenetic rearrangement.

Even though our results are promising, it is very difficult to imagine that the biological clock could be applied to improve vaccination practice. Indeed, a huge body of literature clearly shows that sex and age influence the response to different type of vaccines and yet they are not currently utilized to tailor different vaccination protocols, although they are inexpensive variables compared to DNA methylation analysis. 

However, a personalized medicine perspective can change the picture. Personalized medicine has the aim of developing tailored treatment for individuals or groups of individuals based on their unique characteristics. While personalized vaccination is not yet in practice, it is reasonable to assume that, as personalized medicine becomes more prevalent, it will also impact vaccination strategies. In this view, the application of biological age may prove a useful instrument to identify the individual immune potential if results in the data presented here are corroborated by further studies.

It is worth mentioning that biological age is emerging as a relevant determinant in a vast majority of clinical areas, thus driving interests and efforts for anti-aging interventions and approaches. Our data seem to suggest that, among the benefits of anti-aging therapies (i.e., interventions aimed at decreasing biological age), there could be an improved/restored response to vaccinations. Accordingly, vaccination practice in the near future could benefit from anti-aging approaches at the population level.

## Figures and Tables

**Table 1:** Characteristics of studied cohort. *p*-values were determined by Pearson’s Chi-squared test (for categorical variables) or Welch’s two sample *t*-test (for quantitative variables).

*p*-Value	Non-Responders (NR)	Responders (R)	
-	30	41	Total number of subjects
0.1795	15/15	27/14	Number of Females/Males
0.3333	3.135 × 10^6^ ± 1.705	2.726 × 10^6^ ± 1.791	Average Cell Count ± SD
-	22–62	22–53	Age Range (years)
0.3194	35.92 ± 11.70	33. 45 ± 7.86	Group Average Age (years) ± SD

**Table 2:** Characteristics of studied female and male groups. *p*-values were determined by Welch’s Two Sample *t*-test.

Males (29)			Females (42)			
*p*-Value	NR	R	*p*-Value	NR	R	
-	15	14	-	15	27	Number of subjects
0.2765	3.260 × 10^6 ^ ±1.519	2.632 × 10^6 ^ ±1.522	0.7083	3.010 × 10^6 ^ ±1.918	2.775 × 10^6 ^ ±1.951	Average Cell Count ± SD
-	23–50	26–49	-	22–62	22–53	Age Range [years]
0.8848	37.06 ±9.50	36.64 ±5.76	0.4523	34.80 ±13.81	31.80 ±8.37	Average Age [years] ± SD

**Table 3:** Results of statistical hypothesis test comparing responders and non-responders from overall cohort, using the 2SR approach. *p*-values were calculated with Student’s *t*-test and corrected for multiple testing with BH procedure. Significant *p*-values (<0.05) are underlined.

Adjusted *p*-Value	*p*-Value	Median in Responders	Median in Non-Responders	Number of Outliers	DNAm-Based Estimate
0.081	0.017	26.463	31.792	1	DNAmAge
0.124	0.053	7.226	7.253	2	DNAmTL
0.407	0.349	1.003	1.019	1	DunedinPoAm
0.081	0.023	20.187	25.153	0	AltumAge
0.407	0.318	−28.692	−51.208	1	CRP_CpG_risk_score
0.733	0.733	35.202	35.246	1	PCHorvath1
0.249	0.142	7.449	7.489	3	PCDNAmTL

**Table 4:** Results of statistical hypothesis test comparing female responder and non-responder subjects, using the 2SR approach. *p*-values were calculated with Student’s *t*-test and corrected for multiple testing with BH procedure. Significant *p*-values (<0.05) are underlined.

Adjusted *p*-Value	*p*-Value	Median in Responders	Median in Non-Responders	Number of Outliers	DNAm-Based Estimate
0.045	0.032	24.176	26.727	2	DNAmAge
0.028	0.010	7.229	7.272	1	DNAmTL
0.932	0.932	1.024	0.984	1	DunedinPoAm
0.045	0.030	17.171	19.833	6	AltumAge
0.028	0.006	−29.566	−48.746	2	CRP_CpG_risk_score
0.134	0.115	33.155	28.970	1	PCHorvath1
0.028	0.012	7.457	7.549	1	PCDNAmTL

**Table 5:** Results of statistical hypothesis test comparing male responder and non-responder subjects, using the 2SR approach. *p*-values were calculated with Student’s *t*-test and corrected for multiple testing with BH procedure. Significant *p*-values (<0.05) are underlined.

Adjusted *p*-Value	*p*-Value	Median in Responders	Median in Non-Responders	Number of Outliers	DNAm-Based Estimate
0.730	0.317	32.798	39.082	1	DNAmAge
0.906	0.906	7.225	7.183	0	DNAmTL
0.091	0.013	0.969	1.030	2	DunedinPoAm
0.424	0.121	28.108	36.610	0	AltumAge
0.730	0.625	−37.239	−56.821	0	CRP_CpG_risk_score
0.730	0.599	38.743	41.396	1	PCHorvath1
0.730	0.626	7.397	7.396	1	PCDNAmTL

## Data Availability

The datasets generated and analyzed during the current study are available in the NCBI’s Gene Expression Omnibus [48] and are accessible through GEO Series accession number GSE273657 (https://www.ncbi.nlm.nih.gov/geo/query/acc.cgi?acc=GSE273657).

## References

[1] WHO. World Health Organization Fact Sheets. Hepatitis B. https://www.who.int/news-room/fact-sheets/detail/hepatitis-b.

[2] Mahamat G., Kenmoe S., Akazong E.W., Ebogo-Belobo J.T., Mbaga D.S., Bowo-Ngandji A., Foe-Essomba J.R., Amougou-Atsama M., Monamele C.G., Mikangue C.A.M. (2021). Global prevalence of hepatitis B virus serological markers among healthcare workers: A systematic review and meta-analysis. World J. Hepatol..

[3] Pappas S.C. (2021). Hepatitis B and Health Care Workers. Clin. Liver Dis..

[4] Legge 27 Maggio 1991 n. Legge 27 Maggio 1991, n. 165: Obbligatorietà Della Vaccinazione Contro L’epatite Virale B (Law May 27, 1991, N. 165. Mandatory Vaccination against Viral Hepatitis B). Gazzetta Ufficiale N. 127, 1 June 1991. https://www.gazzettaufficiale.it/eli/id/1991/06/01/091G0201/sg.

[5] Meier M.A., Berger C.T. (2020). A simple clinical score to identify likely hepatitis B vaccination non-responders—Data from a retrospective single center study. BMC Infect. Dis..

[6] Poland G.A., Jacobson R.M. (2004). Prevention of Hepatitis B with the Hepatitis B Vaccine. N. Engl. J. Med..

[7] Hall E. (2021). Epidemiology and prevention of vaccine-preventable diseases. Hepatitis B.

[8] Intesa Governo, Regioni e Province Autonome di Trento e di Bolzano sul “Piano Nazionale di Prevenzione Vaccinale (PNPV) 2023-2025” e sul “Calendario Nazionale Vaccinale”. https://www.trovanorme.salute.gov.it/norme/dettaglioAtto?id=95963&completo=true.

[9] Dentico P., Buongiorno R., Volpe A., Zavoianni A., Pastore G., Schiraldi O. (1992). Long term immunogenicity safety and efficacy of a recombinant hepatitis B vaccine in healthy adults. Eur. J. Epidemiol..

[10] Hess G., Hingst V., Cseke J., Bock H.L. (1992). Clemens R. Influence of vaccination schedules and host factors on antibody response fol-lowing hepatitis B vaccination. Eur. J. Clin. Microbiol. Infect. Dis..

[11] Wood R.C. (1993). Risk Factors for Lack of Detectable Antibody Following Hepatitis B Vaccination of Minnesota Health Care Workers. JAMA.

[12] Vermeiren A.P., Hoebe C.J., Dukers-Muijrers N.H. (2013). High non-responsiveness of males and the elderly to standard hepatitis B vaccination among a large cohort of healthy employees. J. Clin. Virol..

[13] Horvath S. (2013). DNA methylation age of human tissues and cell types. Genome Biol..

[14] Jones M.J., Goodman S.J., Kobor M.S. (2015). DNA methylation and healthy human aging. Aging Cell.

[15] Heyn H., Li N., Ferreira H.J., Moran S., Pisano D.G., Gomez A., Diez J., Sanchez-Mut J.V., Setien F., Carmona F.J. (2012). Distinct DNA methylomes of newborns and centenarians. Proc. Natl. Acad. Sci. USA.

[16] Talens R.P., Christensen K., Putter H., Willemsen G., Christiansen L., Kremer D., Suchiman H.E.D., Slagboom P.E., Boomsma D.I., Heijmans B.T. (2012). Epigenetic variation during the adult lifespan: Cross-sectional and longitudinal data on monozygotic twin pairs. Aging Cell.

[17] Triche T.J., Weisenberger D.J., Van Den Berg D., Laird P.W., Siegmund K.D. (2013). Low-level processing of Illumina Infinium DNA Methylation BeadArrays. Nucleic Acids Res..

[18] Lu A.T., Seeboth A., Tsai P.-C., Sun D., Quach A., Reiner A.P., Kooperberg C., Ferrucci L., Hou L., Baccarelli A.A. (2019). DNA methylation-based estimator of telomere length. Aging.

[19] Belsky D.W., Caspi A., Arseneault L., Baccarelli A.A., Corcoran D.L., Gao X., Hannon E., Harrington H.L., Rasmussed L.J.H., Houts R. (2020). Quantification of the pace of biological aging in humans through a blood test, the DunedinPoAm DNA methylation algorithm. eLife.

[20] Camillo L.P.d.L., Lapierre L.R., Singh R. (2022). A pan-tissue DNA-methylation epigenetic clock based on deep learning. npj Aging.

[21] Wielscher M., Mandaviya P.R., Kuehnel B., Joehanes R., Mustafa R., Robinson O., Zhang Y., Bodinier B., Walton E., Mishra P.P. (2022). DNA methylation signature of chronic low-grade inflammation and its role in cardio-respiratory diseases. Nat. Commun..

[22] Higgins-Chen A.T., Thrush K.L., Wang Y., Minteer C.J., Kuo P.-L., Wang M., Niimi P., Sturm G., Lin J., Moore A.Z. (2022). A computational solution for bolstering reliability of epigenetic clocks: Implications for clinical trials and longitudinal tracking. Nat. Aging.

[23] Di Lello F.A., Martínez A.P., Flichman D.M. (2022). Insights into induction of the immune response by the hepatitis B vaccine. World J. Gastroenterol..

[24] Van Der Meeren O., Crasta P., Cheuvart B., De Ridder M. (2015). Characterization of an age-response relationship to GSK’s recombinant hepatitis B vaccine in healthy adults: An integrated analysis. Hum. Vaccines Immunother..

[25] Weinberger B., Haks M.C., de Paus R.A., Ottenhoff T.H.M., Bauer T., Grubeck-Loebenstein B. (2018). Impaired Immune Response to Primary but Not to Booster Vaccination Against Hepatitis B in Older Adults. Front. Immunol..

[26] Gensous N., Franceschi C., Blomberg B.B., Pirazzini C., Ravaioli F., Gentilini D., Di Blasio A.M., Garagnani P., Frasca D., Bacalini M.G. (2018). Responders and non-responders to influenza vaccination: A DNA methylation approach on blood cells. Exp. Gerontol..

[27] Oh H., Wang S.C., Prahash A., Sano M., Moravec C.S., Taffet G.E., Michael L.H., Youker K.A., Entman M.L., Schneider M.D. (2003). Telomere attrition and Chk2 activation in human heart failure. Proc. Natl. Acad. Sci. USA.

[28] Sampson M.J., Winterbone M.S., Hughes J.C., Dozio N., Hughes D.A. (2006). Monocyte Telomere Shortening and Oxidative DNA Damage in Type 2 Diabetes. Diabetes Care.

[29] Honig L.S., Kang M.S., Schupf N., Lee J.H., Mayeux R. (2012). Association of Shorter Leukocyte Telomere Repeat Length With Dementia and Mortality. Arch. Neurol..

[30] Watfa G., Dragonas C., Brosche T., Dittrich R., Sieber C.C., Alecu C., Benetos A., Nzietchueng R. (2011). Study of telomere length and different markers of oxidative stress in patients with Parkinson’s disease. J. Nutr. Health Aging.

[31] Chin K., de Solorzano C.O., Knowles D., Jones A., Chou W., Rodriguez E.G., Kuo W.-L., Ljung B.-M., Chew K., Myambo K. (2004). In situ analyses of genome instability in breast cancer. Nat. Genet..

[32] Ferrucci L., Gonzalez-Freire M., Fabbri E., Simonsick E., Tanaka T., Moore Z., Salimi S., Sierra F., de Cabo R. (2019). Measuring biological aging in humans: A quest. Aging Cell.

[33] Zhang Z., Reynolds S.R., Stolrow H.G., Chen J., Christensen B.C., Salas L.A. (2023). Deciphering the role of immune cell composition in epigenetic age acceleration: Insights from cell-type deconvolution applied to human blood epigenetic clocks. Aging Cell.

[34] Batliwalla F., Damle R., Metz C., Chiorazzi N., Gregersen P. (2001). Simultaneous flow cytometric analysis of cell surface markers and telomere length: Analysis of human tonsilar B cells. J. Immunol. Methods.

[35] Son N.H., Joyce B., Hieatt A., Chrest F.J., Yanovski J., Weng N.-P. (2003). Stable telomere length and telomerase expression from naı̈ve to memory B-lymphocyte differentiation. Mech. Ageing Dev..

[36] Yusipov I., Bacalini M.G., Kalyakulina A., Krivonosov M., Pirazzini C., Gensous N., Ravaioli F., Milazzo M., Giuliani C., Vedunova M. (2020). Age-related DNA methylation changes are sex-specific: A comprehensive assessment. Aging.

[37] Saco T.V., Strauss A.T., Ledford D.K. (2018). Hepatitis B vaccine nonresponders Possible mechanisms and solutions. Ann. Allergy Asthma Immunol..

[38] Walayat S., Ahmed Z., Martin D., Puli S., Cashman M., Dhillon S. (2015). Recent advances in vaccination of non-responders to standard dose hepatitis B virus vaccine. World J. Hepatol..

[39] Yanny B., Konyn P., Najarian L.M., Mitry A., Saab S. (2019). Management Approaches to Hepatitis B Virus Vaccination Nonresponse. Gastroenterol. Hepatol..

[40] Wu T.-W., Chou C.-L., Chen C.-F., Wang L.-Y. (2023). Common Genetic Variants of Response to Hepatitis B Vaccines Correlate with Risks of Chronic Infection of Hepatitis B Virus: A Community-Based Case-Control Study. Int. J. Mol. Sci..

[41] Wu T., Chen C., Lai S., Lin H.H., Chu C., Wang L. (2015). SNP rs7770370 in *HLA*-*DPB1* loci as a major genetic determinant of response to booster hepatitis B vaccination: Results of a genome-wide association study. J. Gastroenterol. Hepatol..

[42] Png E., Thalamuthu A., Ong R.T., Snippe H., Boland G.J., Seielstad M. (2011). A genome-wide association study of hepatitis B vaccine response in an Indonesian population reveals multiple independent risk variants in the HLA region. Hum. Mol. Genet..

[43] Pan L., Zhang L., Zhang W., Wu X., Li Y., Yan B., Zhu X., Liu X., Yang C., Xu J. (2013). A genome-wide association study identifies polymorphisms in the HLA-DR region associated with non-response to hepatitis B vaccination in Chinese Han populations. Hum. Mol. Genet..

[44] Nishida N., Sugiyama M., Sawai H., Nishina S., Sakai A., Ohashi J., Khor S., Kakisaka K., Tsuchiura T., Hino K. (2018). Key HLA-DRB1-DQB1 haplotypes and role of the BTNL2 gene for response to a hepatitis B vaccine. Hepatology.

[45] Chung S., Roh E.Y., Park B., Lee Y., Shin S., Yoon J.H., Song E.Y. (2019). GWAS identifying *HLA-DPB1* gene variants associated with responsiveness to hepatitis B virus vaccination in Koreans: Independent association of *HLA-DPB1*04:02* possessing rs1042169 G - rs9277355 C - rs9277356 A. J. Viral Hepat..

[46] Davila S., Froeling F.E.M., Tan A., Bonnard C., Boland G.J., Snippe H., Hibberd M.L., Seielstad M. (2010). New genetic associations detected in a host response study to hepatitis B vaccine. Genes Immun..

[47] Ryckman K.K., Fielding K., Hill A.V., Mendy M., Rayco-Solon P., Sirugo G., van der Sande M.A., Waight P., Whittle H.C., Hall A.J. (2010). Host Genetic Factors and Vaccine-Induced Immunity to HBV Infection: Haplotype Analysis. PLoS ONE.

[48] Edgar R., Domrachev M., Lash A.E. (2002). Gene Expression Omnibus: NCBI gene expression and hybridization array data repository. Nucleic Acids Res..

